# Antigen-labeled mesoporous silica-coated Au-core Pt-shell nanostructure: a novel nanoprobe for highly efficient virus diagnosis

**DOI:** 10.1186/s13036-019-0220-1

**Published:** 2019-11-14

**Authors:** Aiyun Li, Lin Long, Fengshou Liu, Jianbo Liu, Xiaochun Wu, Yinglu Ji

**Affiliations:** 1 College of Opto-electronic Engineering, Zaozhuang University, Zaozhuang, 277160 China; 2 Zaozhuang Municipal Center for Disease Control and Prevention, Zaozhuang, 277100 China; 3 CAS Key Laboratory of Standardization and Measurement for Nanotechnology, National Center for Nanoscience and Technology, Beijing, 100190 China

**Keywords:** Gold nanorods, Platinum, Mesoporous silica, Nanozyme, Enzyme-linked immunosorbent assay (ELISA), Virus diagnosis

## Abstract

**Background:**

As an emerging research area of artificial enzymes, nanozyme, the catalytic nanomaterials with enzyme-like characteristics, have attracted enormous attention in research. Here, a nanozyme probe has been realized by utilizing antigen-labeled mesoporous silica-encapsulated Au-core Pt-shell (Au@Pt@SiO_2_) nanostructures for the diagnosis of rubella virus (RV). Pt nanoparticles have been suggested to act as potent peroxidase mimetics with high activities. However, smaller Pt nanoparticles are very easily aggregated, which has negative effects on the catalytic activities.

**Results:**

In this work, the use of gold nanorod as the support favours the well dispersion of the small Pt nanoparticles to improve the stability of them. Furthermore, the designed the silica shell could also isolate the recognition antigens from the surface reactive sites, retaining catalytic activity of the inner nanozyme. In addition, compared with antigen-labeled horseradish peroxidase (HRP), the antigen-labeled Au@Pt@SiO_2_ nanozyme was more stable and robust. A capture enzyme-linked immunosorbent assay (ELISA) for the determination of RV showed that the antigen-labeled Au@Pt@SiO_2_ nanozyme-based ELISA exhibited good sensitivity.

**Conclusions:**

The highly sensitive peroxidase-like activity of antigen-labeled Au@Pt@SiO_2_ nanozyme, along with their catalytic stability and robustness, can facilitate their utilization in biochemical assays and clinical diagnosis.

## Background

The human pathogenic rubella virus (RV) is the cause of German measles, a highly contagious childhood airborne disease that is endemic throughout the world. Rubella infection during pregnancy causes congenital rubella syndrome, including the classic triad of cataracts, cardiac abnormalities and sensorineural deafness [1, 2]. For this reason, it is important to use the most sensitive and efficient detection method for rubella virus. Among the conventional detection methods, rubella immunoglobulin (Ig) M serological testing is a standard method for confirming acute rubella infection [3, 4]. Peroxidases such as HRP are widely applied in enzyme-linked immunosorbent assay (ELISA) to trace the antigen, antibody, virus or cell. However, the instability of HRP can cause a high rate of false-negative results. Thus, developing stable enzyme mimetics is highly appealing [5, 6]. Nanostructures possess an intrinsic enzyme-like activity, catalysing enzyme substrates, which is similar to that of natural enzymes. This type of catalytic inorganic nanomaterial has been termed a nanozyme [7, 8]. Compared with natural enzymes, nanozyme are advantageous in several aspects, such as their low cost, ease of mass production, robustness in harsh environments, high stability, long-term storage ability and large surface area for further modification and bioconjugation [9, 10]. Due to their high stability and easy surface modification, nanozyme with peroxidase-like activity have emerged as alternatives to HRP in immunoassay [11, 12].

As a super catalyst, Pt nanoparticles (NPs) have been extensively explored for applications in fuel cells, hydrogenation, and air purification [13, 14]. Additionally, small Pt NPs have been suggested to act as potent catalase mimetics or peroxidase mimetics, as they can effectively scavenge H_2_O_2_ or catalyse the H_2_O_2_-mediated oxidation of peroxidase substrates [15]. However, the low stability of unsupported Pt NPs under different conditions causes a serious decline in their performance during catalytic operation. A support is often needed to keep them in a well-dispersed state [16, 17]. Previously, we developed a procedure to grow small Pt nanodots on gold nanorods (NRs) and form a rod-shaped Au core/Pt nanodot shell nanostructure. Pt nanodots distribute homogeneously on the surface of the Au rod. Such a structure is highly desirable for catalysis due to its large surface area covered in small Pt nanodots [18]. Furthermore, to be a substitute for an enzyme such as HRP and used in bioassays, nanozyme should have versatile chemistry for further functionalization. However, surface modification always shields the surface active sites of a nanozyme. In recent years, various porous shells have been prepared to encapsulate metal nanoparticles, isolating the active cores and providing convenient channels for chemical species to reach the surface of the active nanoparticles [19, 20]. In particular, the use of mesoporous silicas for protein analysis is a very interesting research field due to their attractive properties such as high surface area, uniform pore size, large pore volume, controllable morphology, high thermal stability, and facile surface functionalization [21, 22]. Additionally, the shell is always chemically inert; thus, the encapsulated nanozyme could have good dispersion stability in PBS buffers or after the addition of chromogenic substrates [23, 24].

**Fig. 1:**
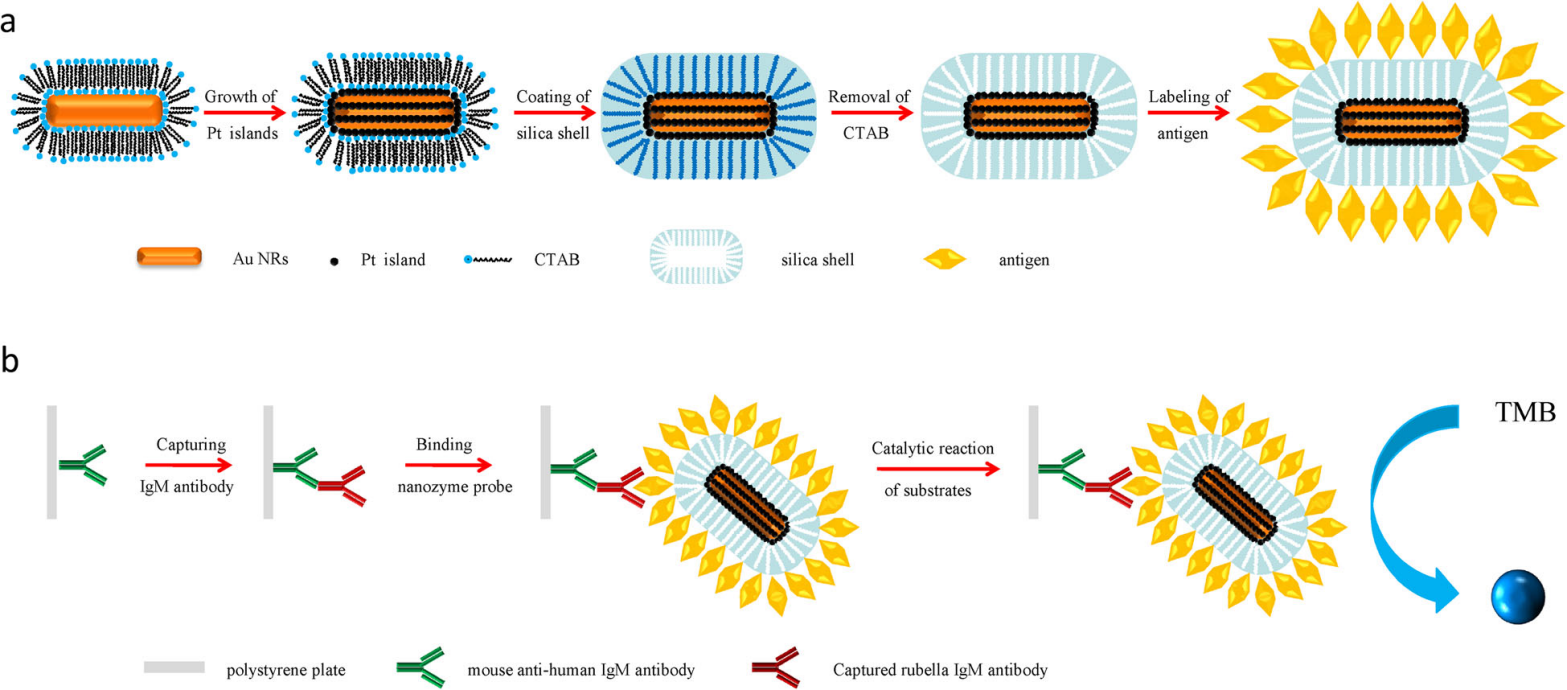
**a** Schematic representation of the synthetic procedure for antigen-labeled Au@Pt@SiO_2_ nanozyme. **b** The illustrated process of the immunoassay of antigen-labeled Au@Pt@SiO_2_ nanozyme based ELISA system

Inspired by mesoporous silica-coated nanocrystals, which reserve the properties of the functional core and are favourable for surface functionalization, herein, we develop a novel Au-core@Pt-shell@mesoporous silica (Au@Pt@SiO_2_) nanozyme for immunoassays. The preparation procedure for the Au@Pt@SiO_2_ nanozyme is described in Fig. 1a. The as-synthesized Au@Pt@SiO_2_ nanozyme are able to catalyse colour reactions in the immunoassay and, therefore, can be used to replace natural enzymes in a conventional ELISA. Then, we designed a novel conjugate based on antigen-labeled Au@Pt SiO_2_ nanozyme, which was used as nanoprobe for virus serodiagnosis. Using captured-type immunoassays, we demonstrate the applicability of an antigen-labeled Au@Pt@SiO_2_ nanozyme for the ultrasensitive colorimetric detection of rubella IgM antibodies (Fig. 1b).

## Results and discussion

### Characterization of au@Pt@SiO_2_ nanozyme and antigen-labeled au@Pt@SiO_2_ nanozyme

**Fig. 2:**
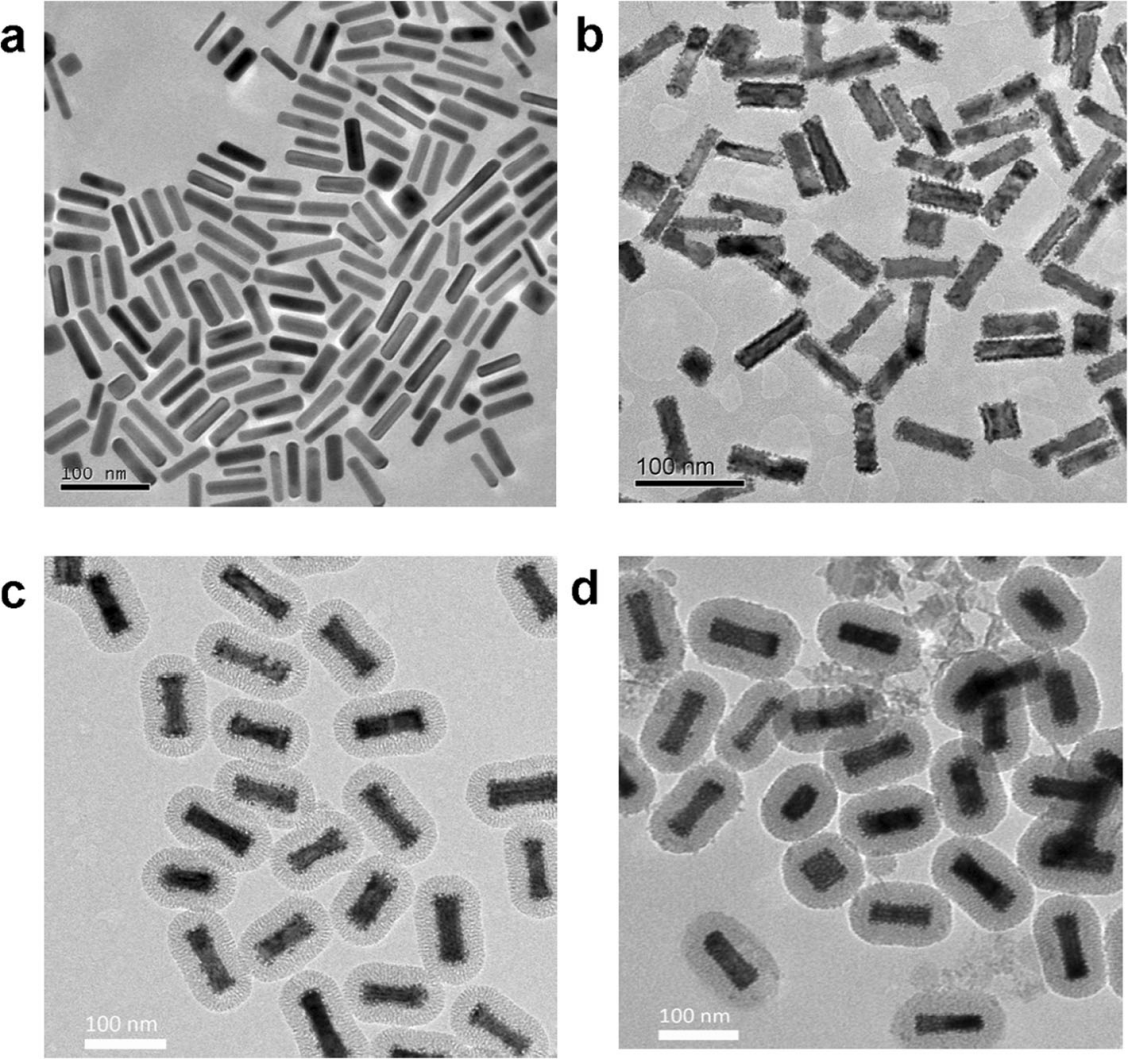
Typical TEM images of (**a**) Au NRs, (**b**) Au@Pt NRs, (**c**) Au@Pt@SiO_2_ nanozyme and (**d**) antigen-labeled Au@Pt@SiO_2_ nanozyme

Au NRs were employed as templates to guide the growth of Pt. The average aspect ratio (AR) of the Au NRs is 3.8 (Fig. 2a). The Pt shows an island growth mode on the Au rod with a Pt/Au ratio of 0.3. Pt nanodots with sizes of 3~4 nm cover the Au rod homogeneously and form a core–shell structure as seen from the TEM image (Fig. 2b), and such a structure is desired for better catalytic activity. The outer mesoporous silica shell is constructed via a surfactant-templating sol-gel approach by using hexadecyltrimethylammonium bromide (CTAB) surfactant as a template. The mesoporous silica layer with a thickness of 25 nm is uniformly coated on the surface of Au@Pt NRs to obtain the Au@Pt@SiO_2_ nanozyme (Fig. 2c). After labeling the Au@Pt@SiO_2_ nanozyme with the rubella antigen, the Au@Pt@SiO_2_ nanozyme still have a uniform morphology and are well-dispersed, and the mesoporous silica shells still present radial channels and ordered nanostructures, as revealed in the TEM image (Fig. 2d).

**Fig. 3:**
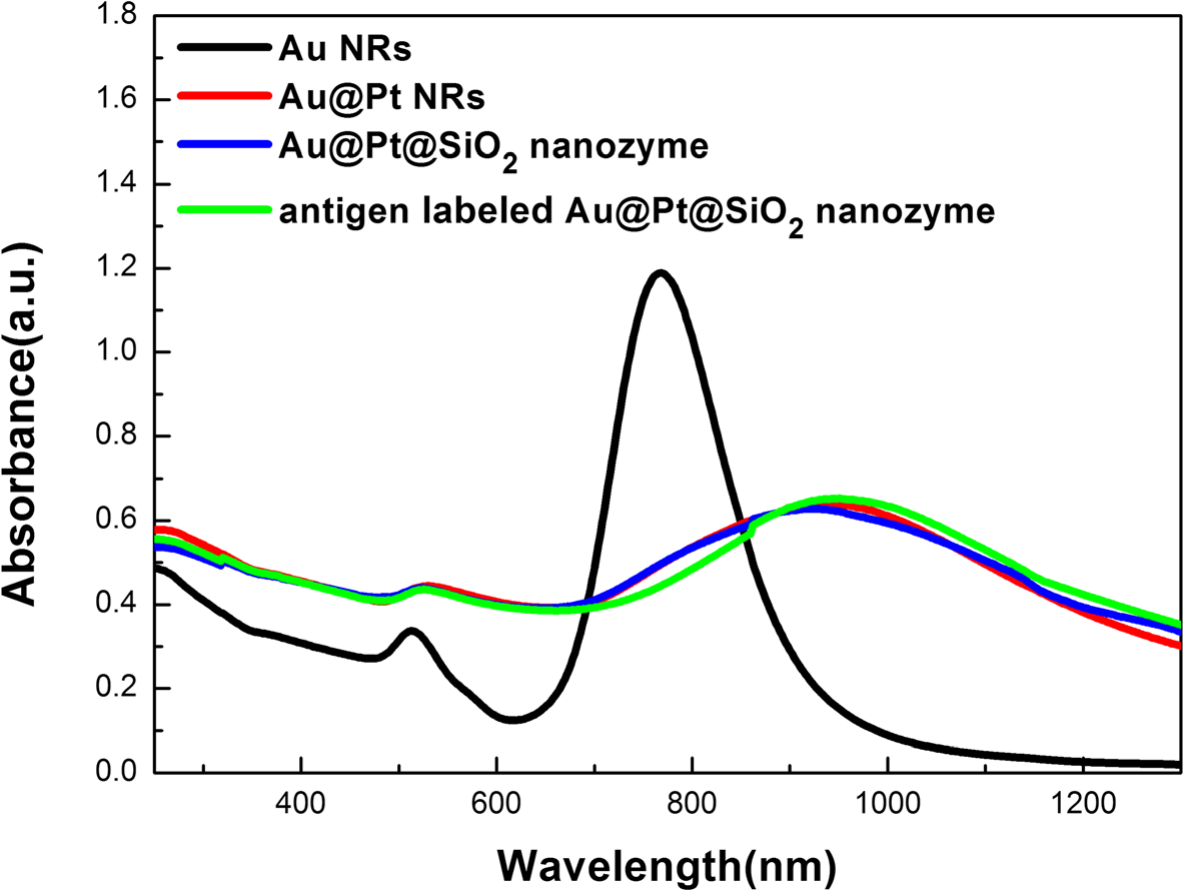
UV-vis-NIR spectra of Au NRs, Au@Pt NRs, Au@Pt@SiO_2_ nanozyme and antigen-labeled Au@Pt@SiO_2_ nanozyme

As shown in Fig. 3, the Au NRs with an AR of 3.8 exhibit a strong longitudinal surface plasmonic resonance (SPR) band with a peak at 780 nm and a weak transverse one peaking at 510 nm. Au@Pt NRs exhibit well-defined and redshifted longitudinal SPR bands in the visible and near-infrared regions. Both the amount and the thickness of Pt determine its contribution to the final position and the strength of the overall SPR features. Upon depositing Pt at a Pt/Au ratio of 0.3, these two bands redshift to 870 nm and 518 nm, respectively. As shown in Fig. 3, the coating of mesoporous silica shell and labeling rubella antigen does not lead to an obvious change in the SPR features of Au@Pt NRs.

To verify the successful preparation of the antigen-labeled Au@Pt@SiO_2_ nanozyme, we conducted dynamic light scattering (DLS) measurements to determine the hydrodynamic diameters of the various nanostructures (Table 1). It is worth mentioning that the DLS analysis assumes that the particles are spherical; hence, due to the rod shape, the diameter from DLS measurements is not the actual size of the NRs. For this reason, the effective diameter is used to evaluate the relative size upon the variation of coatings. Forming a Pt nanodo shell on AuNRs would lead to an increase in the effective diameter. Upon further coating with a shell of silica, the effective diameter of the Au@Pt@SiO_2_ NRs reaches 104.1 nm. After removal of the CTAB templates, there is a slight decrease in the effective diameter of the Au@Pt@SiO_2_ NRs. After antigen labeling, as seen in Table 1, the effective diameter of the Au@Pt@SiO_2_ nanoprobe increased evidently from 94.0 nm to 131.9 nm. The increase in size suggested the presence of antigen on the surface of the Au@Pt@SiO_2_ nanozyme.

**Table 1:** Effective diameter and Zeta potential of various nanoparticles obtained from DLS analysis

Material	Effective diameter (nm)	Zeta potential(mV)
AuNRs	18.1 ± 0.7	24.0 ± 0.8
Au@Pt NRs	48.0 ± 0.4	21.7 ± 1.1
Au@Pt@SiO_2_ NRs with CTAB template	104.1 ± 0.8	−23.9 ± 0.6
Au@Pt@SiO_2_ nanozyme	94.0 ± 0.7	−19.8 ± 0.9
Antigen-labeled Au@Pt@SiO_2_ nanozyme	131.9 ± 2.1	−14.2 ± 0.4

Additionally, in this study, the DLS measurements were used to determine the surface potential of the nanostructures as well. The ζ-potentials of the nanostructures are summarized in Table 1. The as-prepared Au NRs and Au@Pt NRs are positively charged (ζ = + 20 mV) due to the presence of a bilayer of CTAB. The negative ζ-potential shows the successful coating of the Au @Pt NRs by a layer of mesoporous silica (ζ = − 20 mV). Then, the positively charged antigens are labeled on the surface of the Au@Pt@SiO_2_ nanozyme through electrostatic interaction without the need of any cross-linkage reagents. From Table 1, surface charges of NRs were found to become less negative after the antigen labeling process, also providing a strong foundation for the successful binding of antigen to the Au@Pt@SiO_2_ nanozyme.

**Fig. 4:**
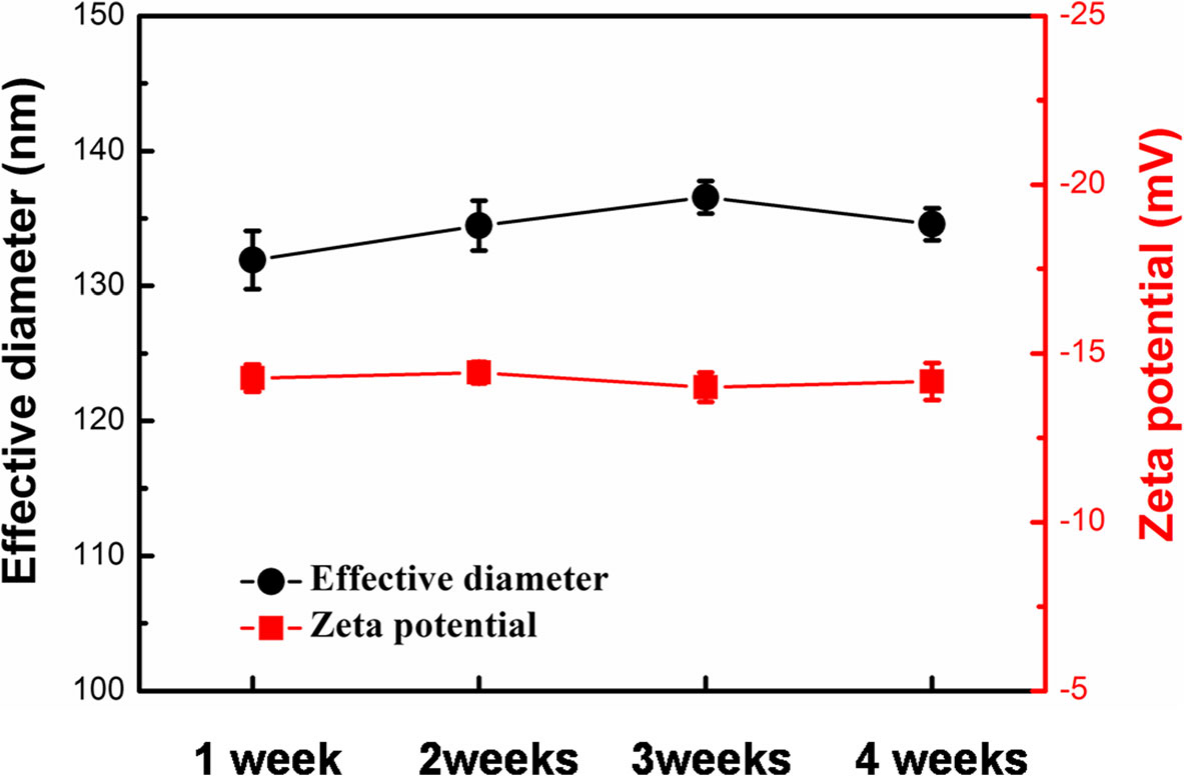
Long-term storage stability of antigen-labeled Au@Pt@SiO_2_ nanozyme in 0.1 M PBS solution (pH = 7.4) at room temperature. All the error bars were calculated based on the standard deviation of three measurements

The stability of antigen-labeled Au@Pt@SiO_2_ nanozyme over storage time was evaluated using zeta potential and their effective diameter (Fig. 4). The antigen-labeled Au@Pt@SiO_2_ nanozyme exhibited a stable average diameter and zeta potential over 4 weeks, further demonstrating their good stability.

### Peroxidase-like activity of antigen-labeled au@Pt@SiO_2_ nanozyme

**Fig. 5:**
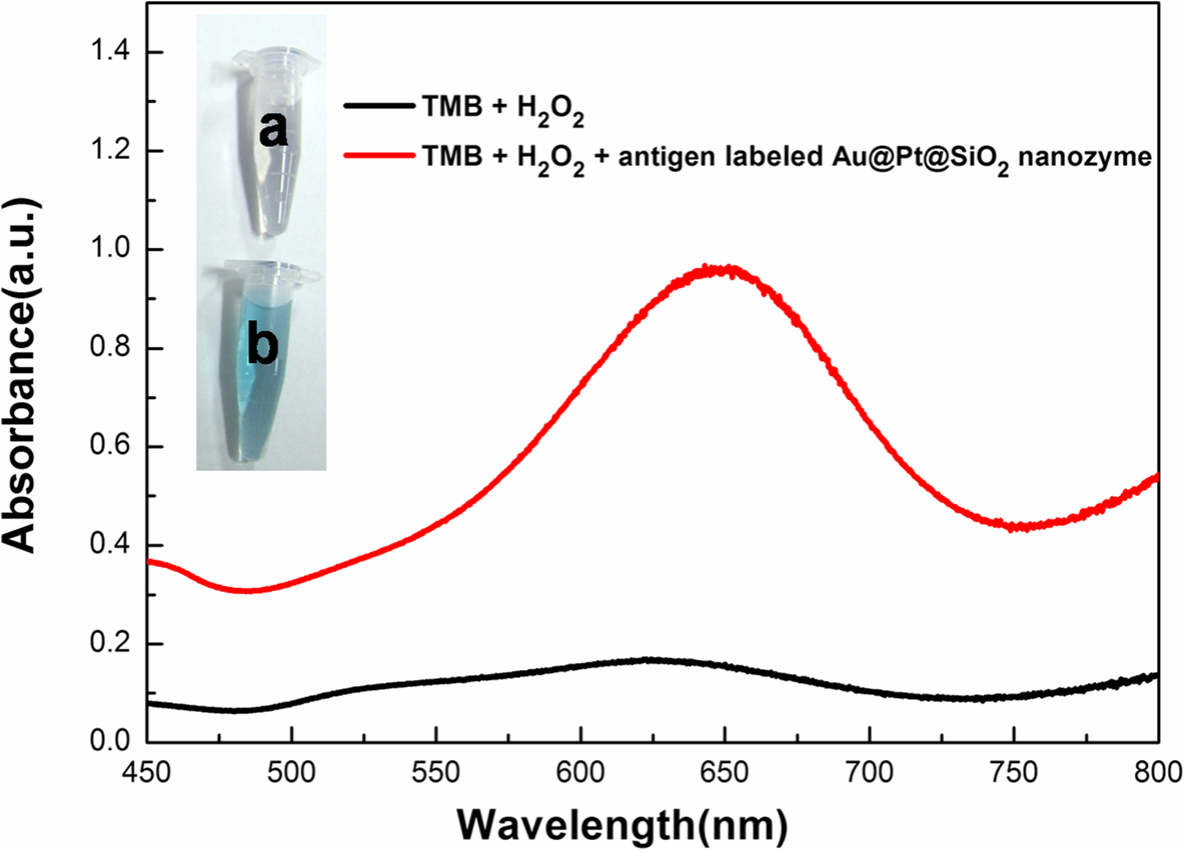
Colour evolution and UV-Vis of catalytical oxidation of TMB. Inset: Photography of the mixture of TMB and H_2_O_2_ in the absence of (a) and in the presence of (b) antigen-labeled Au@Pt@SiO_2_ nanozyme. The corresponding extinction spectra and visual colour changes were recorded after 10 min of incubation

Previously, we found that Au@Pt NRs have intrinsic peroxidase-like activities. In most nanozyme, the binding sites and catalytic sites are not spatially separated; thus, modification and bioconjugation impact the catalytic activities [25]. The encapsulation of Au@Pt NRs in mesoporous silica hindered the interaction between NPs and antigen molecules. That is to say, the mesoporous silica shell kept these active Au@Pt NRs with high enzyme-like catalytic activities while allowing the diffusion of small active molecules in and out of the nanopore channels. We investigated the peroxidase-like activity of antigen-labeled Au@Pt@SiO_2_ nanozyme. 3,3′,5,5′-tetramethylbenzidine (TMB) was employed as a peroxidase substrate for a catalytic oxidation reaction in the presence of H_2_O_2_. There is a characteristic absorption peak at 652 nm with the corresponding development of a blue colour associated with the oxidation of TMB. As shown in Fig. 5, almost no absorption at 652 nm is observed for the TMB-H_2_O_2_ system in the absence of antigen-labeled Au@Pt@SiO_2_ nanozyme. Compared with the TMB-H_2_O_2_ system, the TMB-H_2_O_2_- antigen-labeled Au@Pt@SiO_2_ nanozyme system shows a significant increase in absorbance at 652 nm, indicating that the antigen-labeled Au@Pt@SiO_2_ nanozyme effectively catalyse the oxidation of TMB in the presence of H_2_O_2_. These results clearly demonstrate the intrinsic peroxidase-like property of the antigen-labeled Au@Pt@SiO_2_ nanozyme, which was similar to that of the previously reported Au@Pt nanostructures.

To gain further insight into peroxidase-like behaviour of the antigen-labeled Au@Pt@SiO_2_ nanozyme, we determined the apparent steady-state kinetic parameters for the Au@Pt@SiO_2_ nanozyme and antigen-labeled Au@Pt@SiO_2_ nanozyme towards the H_2_O_2_–TMB catalytic reaction. With the Lineweaver-Burk equation, the Michaelis constant (*K*_m_) and the maximal reaction velocity (*V*_max_) were obtained and shown in Table 2. For natural enzymes, *K*_m_ is an indicator of enzyme affinity to the substrate. A larger *K*_m_ represents a lower affinity whereas a smaller value suggests a higher affinity.

**Table 2:** Apparent kinetic parameters (*K*_m_, *V*_max_) of the Au@Pt@SiO_2_ nanozyme and antigen-labeled Au@Pt@SiO_2_ nanozyme

catalyst	substrate	*K*_m_ (mM)	*V*_max_, (nM·S^− 1^)
Au@Pt@SiO_2_ nanozyme	TMB	0.124	205.5
Antigen labeled Au@Pt@SiO_2_ nanozyme	TMB	0.132	172.3
Au@Pt@SiO_2_ nanozyme	H_2_O_2_	121.8	619.3
Antigen labeled Au@Pt@SiO_2_ nanozyme	H_2_O_2_	111.8	539.0

For TMB substrate, a little increase in *K*_m_ value of the antigen-labeled Au@Pt@SiO_2_ nanozyme was observed (Table 2), suggesting that the antigen-labeled Au@Pt@SiO_2_ nanozyme have a slightly lower affinity for TMB than non-labeled Au@Pt@SiO_2_ nanozyme. This lower affinity may be attributed to the electrostatic interactions between the substrate and the surface of the nanozyme. After the antigen labeling process, the surface charges of NRs were found to become less negative (Table 1), which may decreased the binding affinity between the nanozyme and the positively charged TMB substrate. In contrast to TMB, for H_2_O_2_ the substrate, a decrease in *K*_m_ value was observed for antigen-labeled Au@Pt@SiO_2_ nanozyme since electrostatic interactions might be less important in this case. Notably, the *V*_max_ value of antigen-labeled Au@Pt@SiO_2_ and non-labeled Au@Pt@SiO_2_ nanozyme showed a similar level of activity toward TMB and H_2_O_2_. (The antigen-labeled Au@Pt@SiO_2_ maintained 90% activity of non-labeled Au@Pt@SiO_2_ nanozyme.) Compared to previous report [25], the effect of biomolecules (antigen) showed less significant effect on catalytic activity of Au@Pt@SiO_2_ nanozyme. The little loss of the activity is ascribed to the fact that the fabrication of the silica shell on the Au@Pt NRs. Although the physical presence of the silica shell could affect the diffusion of substrate approaching the surface of the nanozyme, the silica shell could also isolate the antigen from the surface reactive sites, retaining catalytic activity of the inner nanozyme.

### Comparison of catalytic stability of antigen-labeled au@Pt@SiO_2_ nanozyme and antigen-labeled HRP against temperature, pH

**Fig. 6:**
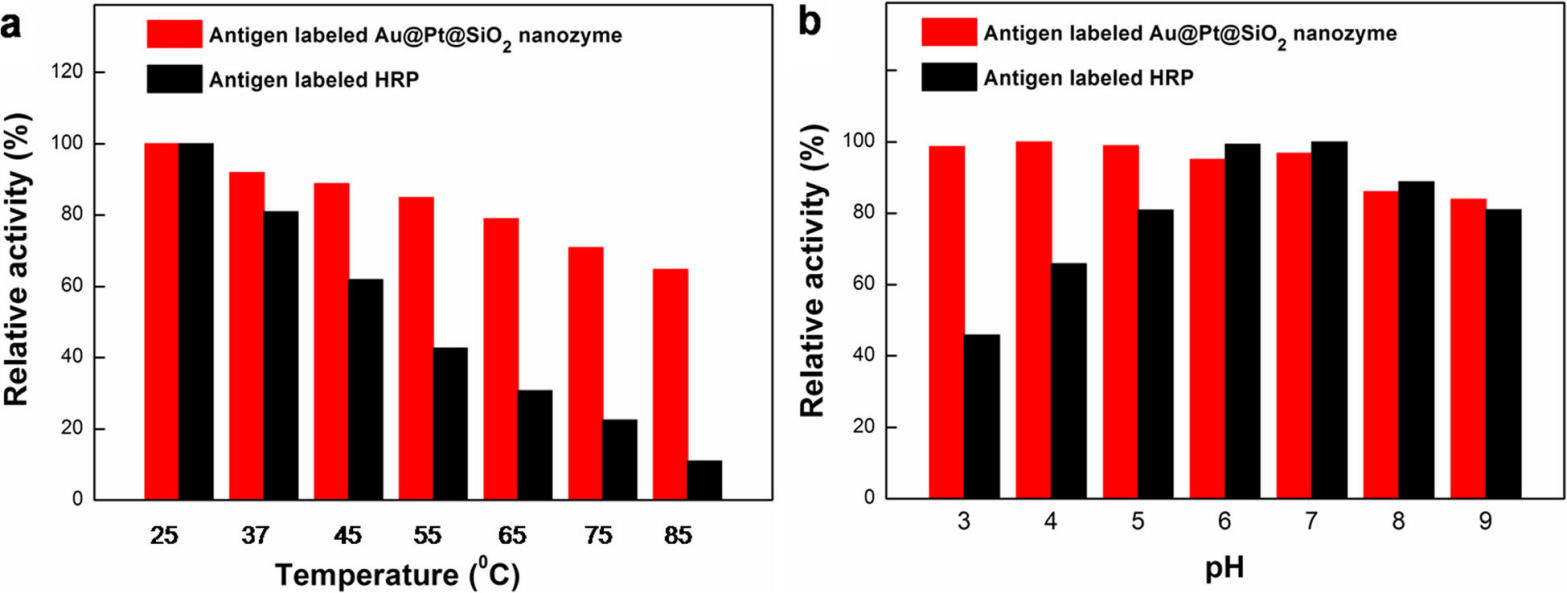
Comparison of the stability of antigen-labeled Au@Pt@SiO_2_ nanozyme and antigen-labeled HRP. **a** antigen-labeled Au@Pt@SiO_2_ nanozyme and antigen-labeled HRP were treated at a wide range of temperatures between 20 and 80 °C for 3 h, and the peroxidase activity was measured under standard conditions. **b** antigen-labeled Au@Pt@SiO_2_ nanozyme and antigen-labeled HRP were treated in media with a range of pH from 3 to 9 for 3 h, and then their peroxidase activities were measured under standard conditions

To further examine the endurance capacity of the antigen-labeled Au@Pt@SiO_2_ nanozyme (i.e., thermal stability and pH tolerance), a comparative study with conventional antigen-labeled HRP was carried out by assaying their catalytic activities towards TMB-H_2_O_2_ under different conditions. Initially, antigen-labeled Au@Pt@SiO_2_ nanozyme or antigen-labeled HRP samples were deposited into solutions with different pH values or temperatures for 3 h, and then the corresponding catalytic activity was measured. As shown in Fig. 6a, the catalytic activity of antigen-labeled Au@Pt@SiO_2_ nanozyme was not much changed over a wide temperature range from 25 to 85 °C, while that of HRP decreased mostly after 45 °C. The reason might be the fact that HRP is a kind of protein and is easily denatured at high temperatures. Furthermore, the catalytic activity of Au@Pt@SiO_2_ nanozyme could be preserved over a wider pH range (6.0–14.0) than that of HRP (6.0–11.0) (Fig. 6b). The silica shell on the Au@Pt@SiO_2_ nanozyme endows this nanozyme probe with good stability in strong acidic solutions or at high temperature.

### Optimization of catalytic conditions of antigen-labeled au@Pt@SiO_2_ nanozyme

**Fig. 7:**
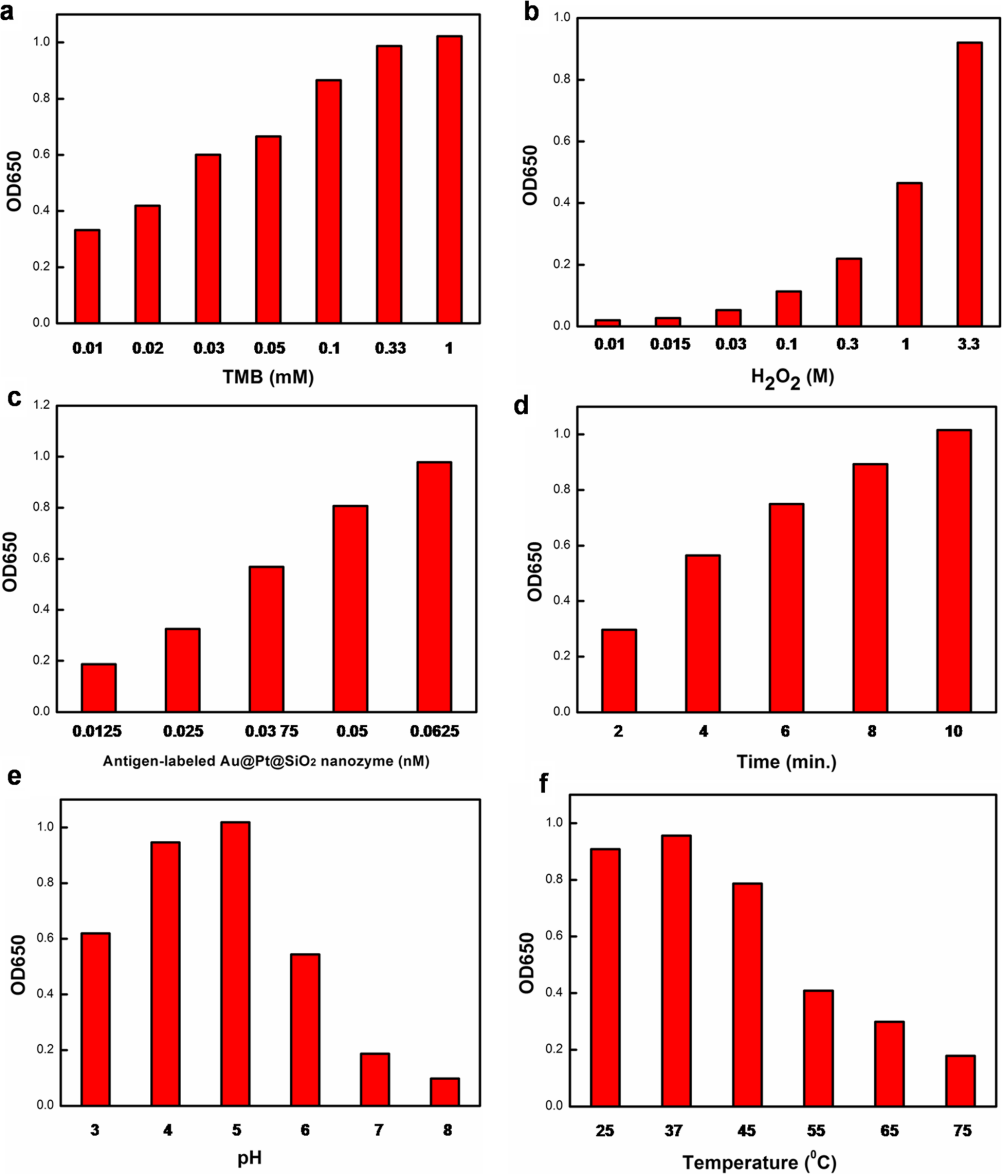
Effects of substrates concentration of TMB, H_2_O_2_, concentration of antigen-labeled Au@Pt@SiO_2_ nanozyme, temperature, reaction time and pH on catalytic activity of the antigen-labeled Au@Pt@SiO_2_ nanozyme. Reaction conditions: (**a**) 0.0625 nM antigen-labeled Au@Pt@SiO_2_ nanozyme, 100 mM H_2_O_2_, (**b**) 0.0625 nM antigen-labeled Au@Pt@SiO_2_ nanozyme and 1 mM TMB, (**c**) 1 mM TMB and 100 mM H_2_O_2_, (**d**-**f**) 0.0625 nM antigen-labeled Au@Pt@SiO_2_ nanozyme, 1 mM TMB and 100 mM H_2_O_2_

Like HRP and other peroxidase mimics, the peroxidase-like activity of the antigen-labeled Au@Pt@SiO_2_ nanozyme is strongly dependent on TMB and H_2_O_2_ concentrations and pH, temperature and other catalytic conditions. For the substrate concentration-dependent activity, the results showed that the highest antigen-labeled Au@Pt@SiO_2_ nanozyme activity could be obtained by adding 0.33 mM TMB (Fig. 7a). Further increasing the concentration of TMB changes the catalytic activity slightly. In contrast, no catalytic activity inhibition was found for the antigen-labeled Au@Pt@SiO_2_ nanozyme-catalysed reaction at an H_2_O_2_ concentration up to 3 M (Fig. 7b). The absorbance at 650 nm showed an almost linear increase with antigen-labeled Au@Pt@SiO_2_ nanozyme concentration from 0.0125~0.0625 nM (Fig. 7c). With increasing reaction time, the curve also increased linearly (Fig. 7d). The pH experiments were performed by using the buffer solution as the reaction media while varying the pH from 3 to 8. The results demonstrated that the efficiency of the catalytic oxidation was much higher in acidic solutions than in neutral solutions (Fig. 7e). The maximum catalytic efficiency occurred at approximately pH 5. The effect of temperature-dependent on the catalytic activity of antigen-labeled Au@Pt@SiO_2_ nanozyme was also studied over a wide temperature range from 25 to 75 °C. The optimal temperature is approximately 37 °C (Fig. 7f), which is consistent with the conventional antigen-labeled HRP.

Based on above these results, we adopted 1 mM TMB, 100 mM H_2_O_2_, 0.0625 nM antigen-labeled Au@Pt@SiO_2_ nanozyme, 10 min, pH 5 and 37 °C as standard conditions for the following biomedical assay.

### Application of biomedical assay

Based on the abovementioned results, the antigen-labeled Au@Pt@SiO_2_ nanozyme were utilized as a nanoprobe for the determination of rubella IgM antibodies. The working principle of the antigen-labeled Au@Pt@SiO_2_ nanozyme for detection of IgM is schematically represented in Fig. 1b. The assay was performed in anti-human IgM antibody-immobilized microplate wells. Diluted test serum was then added and the rubella IgM antibodies present in the serum bound to anti-human IgM antibody. Then, the antigen-labeled Au@Pt@SiO_2_ nanozyme were added, and further incubation was carried out so that the antigen-labeled Au@Pt@SiO_2_ nanozyme were enriched via specific antigen-antibody binding. The unbound antigen-labeled Au@Pt@SiO_2_ nanozyme were removed after washing the plate. Last, the bound antigen-labeled Au@Pt@SiO_2_ nanozyme catalysed the TMB-oxidation reaction and produced a blue colour in the presence of H_2_O_2_, and the absorbance of the oxidation product was monitored at 650 nm.

**Fig. 8:**
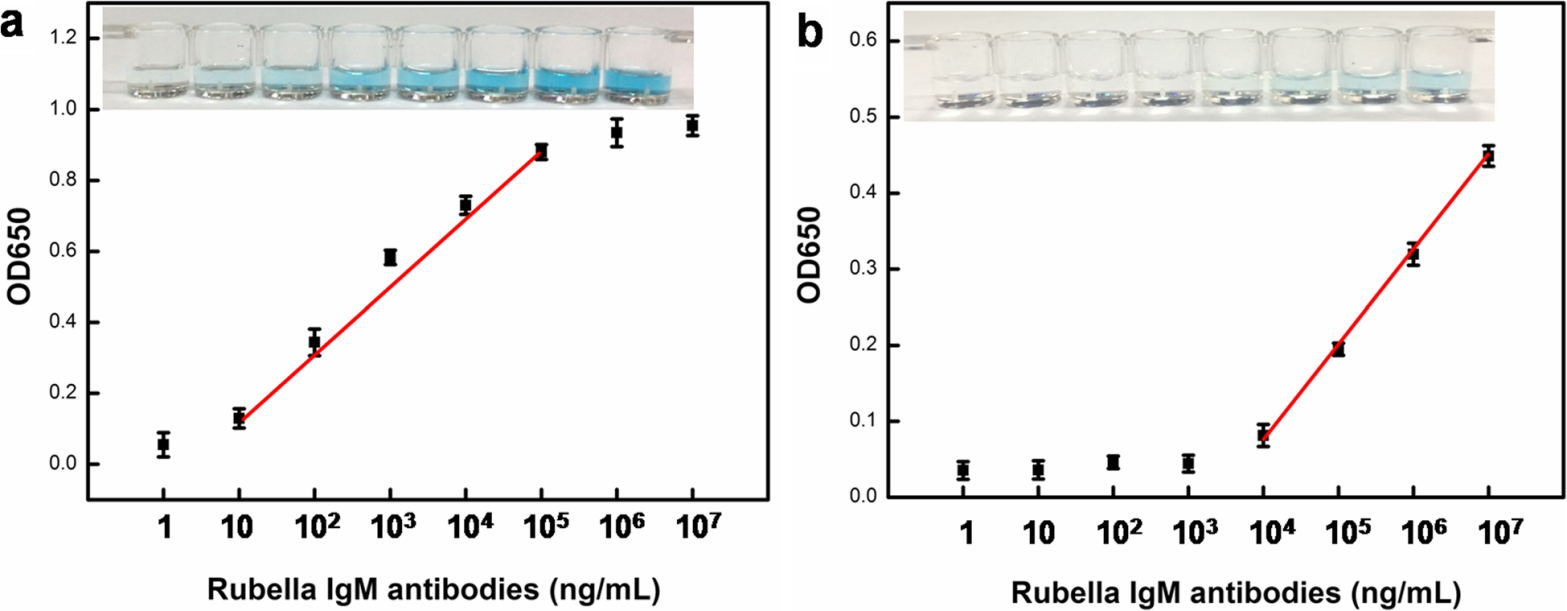
The relation of the mean absorbance intensity at 650 nm and rubella IgM antibodies concentration. **a** antigen-labeled Au@Pt@SiO_2_ nanozyme-based ELISA; (**b**) antigen-labeled HRP-based ELISA. All error bars were calculated based on the standard deviation of three measurements. The insets are the corresponding colour in the well

As shown in Fig. 8a, the absorbance increased with increasing rubella IgM antibody concentrations in the sample. This result was expected, as an increasing concentration of rubella IgM antibodies translates to an increasing amount of antigen-labeled Au@Pt@SiO_2_ nanozyme captured by the formation of sandwich-layered structure. A linear dependence between the absorbance and the rubella IgM antibodies concentration was obtained in the linear range from 10 to 10^5^ ng mL^− 1^, and the detection limit is as low as 10 ng/mL. For comparison, the conventional ELISA protocol was also employed for the detection of rubella IgM antibodies by using antigen-labeled HRP as a signal probe. The linear range was from 10^4^ to 10^7^ ng mL^− 1^ (Fig. 8b). Notably, the used antigen-labeled Au@Pt@SiO_2_ nanozyme show an excellent peroxidase-like catalytic efficiency that is much higher than that of antigen-labeled HRP. The increase in sensitivity attribute to the use of Au@Pt@SiO_2_ nanozyme as an antigen label. First, compared to that of natural HRP enzyme, Au@Pt@SiO_2_ nanozyme provides most catalytic sites, resulting in the strongest peroxidase-like activity. Second, the mesoporous silica shell with high surface areas and large pore volume offer a possibility to load numbers of antigen to the surface of the Au@Pt@SiO_2_ nanozyme, which provides better detection specificity for rubella IgM antibodies.

The reproducibility and precision of the antigen-labeled Au@Pt@SiO_2_ nanozyme-based colorimetric immunoassay are evaluated by calculating the inter- and intra-batch variation coefficients (CVs, *n* = 10). The results are shown in Table 3. The experimental results suggested that the inter-assay and intra-assay CVs were between 5.0 and 14% in all cases. These results revealed that the antigen-labeled Au@Pt@SiO_2_ nanozyme-based colorimetric immunoassays could be used repeatedly and further verified the possibility of batch analysis.

**Table 3:** Inter- and intra-batch variation coefficients of antigen-labeled Au@Pt@SiO_2_ nanozyme-based colorimetric immunoassay

Concentration	10 ng/ml	1μg/ml	0.1 mg/ml
Inter-assay CV (%)	7.19	5.23	7.97
Intra-assay CV (%)	12.3	12.9	14.4

**Fig. 9:**
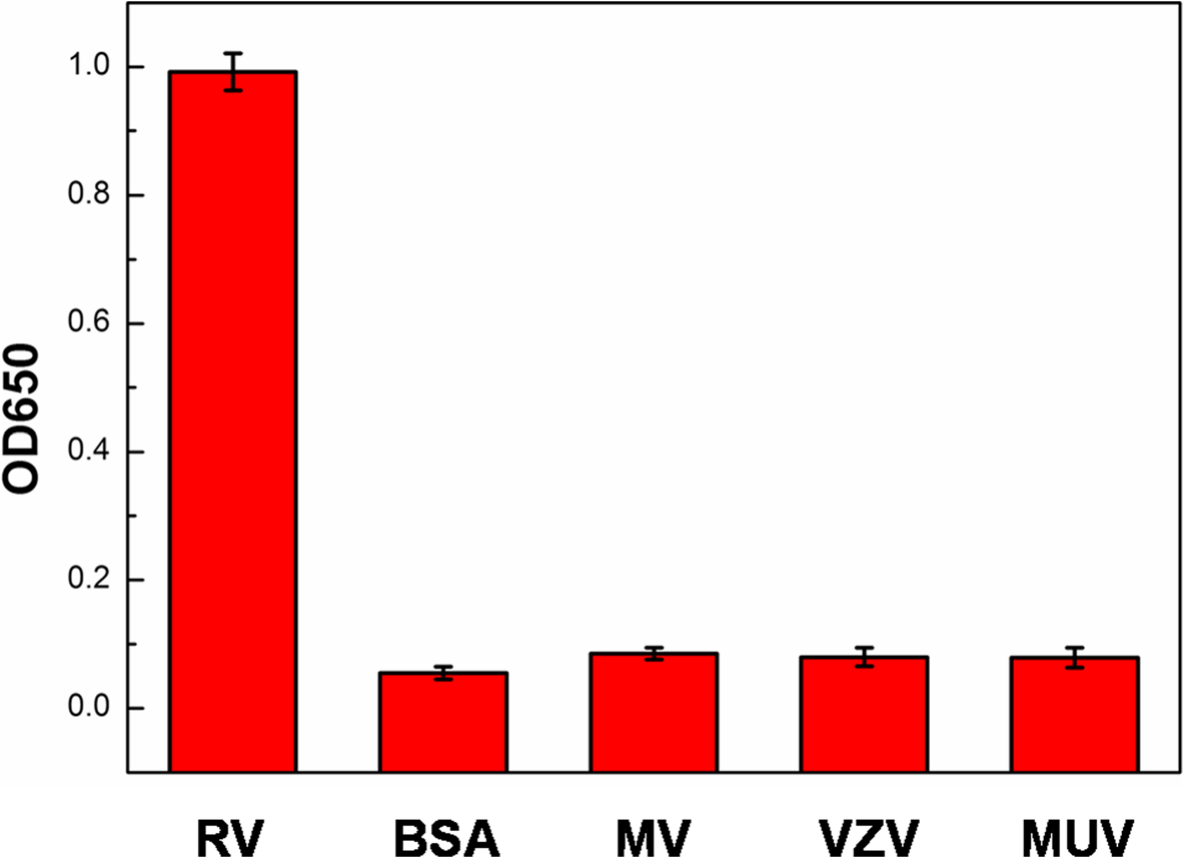
Specificity of RV, measles virus (MV), varicella-zoster virus (VZV) and mumps virus (MUV) positive serum using antigen-labeled Au@Pt@SiO_2_ nanozyme-based ELISA

We chose other infectious viruses, such as measles virus (MV), varicella-zoster virus (VZV) and mumps virus (MUV) IgM antibodies to test the specificity of rubella IgM antibodies detection of this the antigen-labeled Au@Pt@SiO_2_ nanozyme-based colorimetric immunoassays. As shown in Fig. 9, almost no signals are obtained for the other samples, while the absorbance for the RV positive serum is obvious. Therefore, the current sensing method has a high selectivity for rubella IgM antibodies detection.

The results above demonstrated that this system would have excellent capability in response to changes of the clinical serum samples. For clinical serum samples, the standard tests were performed by commercial ELISA, and 20 positive samples and 30 negative samples were employed (Additional file 1: Table. S1). As shown in Table 4, 100% (20/20) of positive clinical samples were detected as positive by the antigen-labeled Au@Pt@SiO_2_ nanozyme-based ELISA, and none of the negative samples were detected as positive by this mmethod, which further proving the good accuracy and reliability of the proposed colorimetric immunoassay for the preliminary detection of rubella IgM antibodies in clinical diagnosis.

**Table 4:** Comparison of assay performance of antigen-labeled Au@Pt@SiO_2_ nanozyme-based ELISA and commercial ELISA for clinical serum samples

Assay	Positive	Negative
Commercial ELISA	20	30
Antigen-labeled Au@Pt@SiO_2_ nanozyme-based ELISA	20	30

## Conclusions

In summary, we developed a novel nanozyme probe for the ultrasensitive detection of rubella IgM antibodies in sera. The rationale of detection is based on the antigen-labeled Au@Pt@SiO_2_ nanozyme. The results demonstrate that antigen-labeled Au@Pt@SiO_2_ nanozyme retained their intrinsic peroxidase-like activity to the same degree as Au@Pt@SiO_2_ nanozyme. Compared with conventional natural enzyme labels, the antigen-labeled Au@Pt@SiO_2_ nanozyme showed the advantages of being low-cost, being easy to prepare, having high peroxidase-like activity and being robust to harsh environments. Based on the enhanced catalytic properties of this nanoenzyme probe, the sensitivity of rubella IgM antibodies is lowered to 10 ng/mL. Hence, this study demonstrates the antigen-labeled Au@Pt@SiO_2_ nanozyme with their superior catalytic activity can be utilized as an alternative to conventional natural enzyme labels for the highly sensitive virus diagnosis in future clinical applications under various conditions.

## Methods

### Material

Sodium borohydride (NaBH_4_), cetylmethylammonium bromide (CTAB), chloroauric acid (HAuCl_4_·3H_2_O), potassium tetrachloroplatinate(II) (K_2_PtCl_4_), silver nitrate (AgNO_3_), sodium hydroxide (NaOH), tetraethyl orthosilicate (TEOS), L-ascorbic acid (AA), 30% H_2_O_2_, and TMB were all purchased from Alfa Aesar (USA) and used as received. The rubella antigen was purchased from Beier Bioengineering Company (China). Rubella antigen, mouse anti-human IgM antibody-coated plate, antigen labelled HRP, and positive and negative serum samples (ELISA kit) were purchased from Kerunda Bioengineering company (Shenzhen, China). Milli-Q water (18 MΩ cm) was used for all solution preparations.

### Synthesis of gold nanorods (au NRs)

Au NRs were synthesized using a seed-mediated growth procedure. CTAB-capped Au seeds were synthesized by chemical reduction of HAuCl_4_ with NaBH_4_. CTAB (7.5 mL, 0.1 M) was mixed with HAuCl_4_ (100 μL, 24 mM), diluted with water to 9.4 mL, and stirred with a magnetic stirrer. Then, ice-cold NaBH_4_ (0.6 mL, 0.01 M) was added. The solution colour immediately turned from bright yellow to brown, indicating the formation of seeds. The Au seeds were used within 2–5 h. A 120 μL aliquot of the seed solution was added to the growth solution consisting of CTAB (100 mL, 0.1 M), HAuCl_4_ (2.04 mL, 24 mM), AgNO_3_ (1.05 mL, 10 mM), H_2_SO_4_ (2 mL, 0.5 M) and AA (800 μL, 0.1 M) to initiate the growth of Au NRs. After 12 h, the reaction was stopped. The obtained Au NRs were purified by centrifuging the solution at 12000 rpm for 5 min twice. The precipitate was collected and re-dispersed in deionized water.

### Synthesis of au@Pt NRs

Au NR solutions (1 mL) were mixed with 62.5 μL of 2 mM PtCl_4_^2−^ aqueous solution. Then, 12.5 μL of 0.1 M AA was added, and the total solution volume was diluted to 2 mL. The mixture was shaken vigorously and then placed in a 30 °C water bath for 30 min. Within several minutes, the colour of the solution changed from pink-red to dark grey, suggesting the formation of a Pt shell. Then, 1 mL of 0.1 M CTAB was added. The obtained Au@Pt NRs were purified by centrifuging the solution at 12,000 rpm for 5 min twice. The precipitate was collected and re-dispersed in deionized water.

### Preparation of au@Pt@SiO_2_ nanozyme

The as-synthesized Au@Pt NRs were dispersed in a mixture containing 10 mL of water, 75 μL of 0.1 M CTAB and 50 μL of 0.2 M NaOH and stirred at 30 °C. Three 30 μL aliquots of 20% TEOS in ethanol were subsequently added under gentle stirring at 30 min intervals. The mixture was incubated for 24 h at 30 °C. The samples were purified by centrifuging the solution at 9500 rpm for 10 min twice. The precipitate was collected and dispersed in 60 mL of NH_4_NO_3_/ethanol solution (6 g/L) for 24 h at 50 °C, and then centrifuged and washed with ethanol twice to remove the CTAB template to obtain Au@Pt@SiO_2_ nanozyme.

### Preparation of au@Pt@SiO_2_ nanozyme

The as-synthesized Au@Pt NRs were dispersed in a mixture containing 10 mL of water, 75 μL of 0.1 M CTAB and 50 μL of 0.2 M NaOH and stirred at 30 °C. Three 30 μL aliquots of 20% TEOS in ethanol were subsequently added under gentle stirring at 30 min intervals. The mixture was incubated for 24 h at 30 °C. The samples were purified by centrifuging the solution at 9500 rpm for 10 min twice. The precipitate was collected and dispersed in 60 mL of NH_4_NO_3_/ethanol solution (6 g/L) for 24 h at 50 °C, and then centrifuged and washed with ethanol twice to remove the CTAB template to obtain Au@Pt@SiO_2_ nanozyme.

### Preparation of antigen-labeled au@Pt@SiO_2_ nanozyme

The as-synthesized Au@Pt@SiO_2_ nanozyme solution (50 μL, 5 nM) was first dispersed into 1 mL of PBS buffer (0.1 M, pH 7.4). Then, 50 μL of 10 mg/mL rubella antigen was added to the above Au@Pt NRs solution and incubated at 4 °C for 96 h. Then, to remove the excess antigen, it was centrifuged at 12,000 r/min for 5 min twice. The clear supernatant was carefully removed, and the precipitate was collected and re-dispersed in 100 μL of PBS buffer (0.1 M, pH 7.4).

### Kinetic analysis

The apparent kinetic parameters were obtained by using the Lineweaver-Burk double reciprocal plot:

$$ \frac{1}{\mathrm{v}}=\left(\frac{K_m}{V_{\mathrm{max}}}\right)\frac{1}{\left[c\right]}+\frac{1}{V_{\mathrm{max}}} $$

where v is the initial velocity, *V*_max_ is the maximal reaction velocity, and [c] is the concentration of substrate.

The reaction kinetics for the catalytic oxidation of TMB in the presence of H_2_O_2_were studied by recording the absorption spectra at 0.25 min intervals using a Varian Cary 50 in kinetics mode. Steady-state kinetic assays were carried out at 37 °C in 0.1 M PBS buffer (pH 5) in the presence of NRs (0.0625 nM). For TMB as the substrate, the H_2_O_2_ concentration was fixed at 100 mM. For H_2_O_2_ as the substrate, the TMB concentration was fixed at 0.5 mM.

### Detection of rubella IgM antibodies by ELISA

ELISA detection of rubella IgM antibodies was performed in 96-well polystyrene plates. Each well of the 96-well plates was pre-coated with mouse anti-human IgM antibodies. First, each well was blocked with 5% BSA (diluted in PBS, pH 7.4) for 1 h at 37 °C to avoid non-specific interaction with the plate surface. Then, the plates were washed five times with PBST buffer (pH 7.4). After that, 100 μl of negative control, positive control or diluted sample was added to the plate and incubated at 37 °C for 1 h. The plates were washed five times with PBST buffer (pH 7.4) to remove the unbound rubella IgM antibodies. Then, 100 μl of antigen-labeled Au@Pt@SiO_2_ nanozyme was added to each well and incubated for 0.5 h at 37 °C. The plates were washed five times with PBST buffer (pH 7.4) to remove the unbound antigen-labeled Au@Pt@SiO_2_ nanozyme. The colour development was initiated by adding 100 μL of substrate solution (1 mM TMB, 100 mM H_2_O_2_ in PBS buffer, pH 5) into each well. After 10 min, absorbance was measured at 650 nm. The clinical serum sample was selected from patients with clinical signs of rubella, or patients who had been exposed to rubella. For the performance of the assay, the clinical serum samples have to be diluted 1:100 with sample diluent. The clinical serum experiment was checked with the positive control, negative control and the blank. Buffer solution was used as the blank.

### Characterizations

UV-vis-NIR extinction spectra were obtained from a Varian Cary 50. Transmission electron microscopy (TEM) was performed on a Tecnai G2 T20 S-TWIN (T20). The zeta potential data were obtained from a Delsa Nano C (Beckman Coulter). ELISA data was obtained on an Infinite™ M200.

## Supplementary information

**Additional file 1: Table S1.** Detection of rubella IgM in the clinical serum obtained by the antigen-labeled Au@Pt@SiO_2_ nanozyme-based ELISA.

## Data Availability

All data generated or analyzed during this study are included in the article and Additional file.
