# Supplementary material for: Antigen-labeled mesoporous silica-coated Au-core Pt-shell nanostructure: a novel nanoprobe for highly efficient virus diagnosis
Source: J Biol Eng. 2019 Nov 14;13:87. doi: 10.1186/s13036-019-0220-1 (PMC6857339; doi:10.1186/s13036-019-0220-1)
Supplement: Supplementary file 1 — Additional file 1: Table S1. Detection of rubella IgM in the clinical serum obtained by the antigen-labeled Au@Pt@SiO2 nanozyme-based ELISA. [file 13036_2019_220_MOESM1_ESM.docx]

**Additional file 1**

**Antigen-labeled mesoporous silica-coated Au-core Pt-shell nanostructure: a novel nanoprobe for highly efficient virus diagnosis**

**Aiyun Li^a,†^, Lin Long^a,b,†^, Fengshou Liu^a^, Jianbo Liu^a,^*, Xiaochun Wu^c,^*, Yinglu Ji^c^**

*^a^ College of Opto-electronic Engineering, Zaozhuang University, Zaozhuang 277160, China*

*^b^ Zaozhuang Municipal Center for Disease Control and Prevention, Zaozhuang 277100, China*

*^c^CAS Key Laboratory of Standardization and Measurement for Nanotechnology, National Center for Nanoscience and Technology, Beijing 100190, China*

*^†^These authors contributed equally to this work*

** Corresponding author email: linyibm@163.com (J. Liu), wuxc@nanoctr.cn (X. Wu))*

**Table S1** **Detection of rubella IgM in the clinical serum obtained by the antigen-labeled Au@Pt@SiO_2_ nanozyme-based ELISA.**

| **Sample** | **OD650** | **Result (Positive / Negative)** |
| --- | --- | --- |
| 1 | 0.0735 | Negative |
| 2 | 0.0364 | Negative |
| 3 | 0.0601 | Negative |
| 4 | 0.054 | Negative |
| 5 | 0.0473 | Negative |
| 6 | 0.0445 | Negative |
| 7 | 0.0548 | Negative |
| 8 | 0.0573 | Negative |
| 9 | 0.053 | Negative |
| 10 | 0.0703 | Negative |
| 11 | 0.0304 | Negative |
| 12 | 0.0587 | Negative |
| 13 | 0.0573 | Negative |
| 14 | 0.0565 | Negative |
| 15 | 0.0435 | Negative |
| 16 | 0.057 | Negative |
| 17 | 0.0487 | Negative |
| 18 | 0.0532 | Negative |
| 19 | 0.0435 | Negative |
| 20 | 0.0344 | Negative |
| 21 | 0.0456 | Negative |
| 22 | 0.064 | Negative |
| 23 | 0.0714 | Negative |
| 24 | 0.0367 | Negative |
| 25 | 0.0386 | Negative |
| 26 | 0.048 | Negative |
| 27 | 0.0502 | Negative |
| 28 | 0.0436 | Negative |
| 29 | 0.0792 | Negative |
| 30 | 0.0453 | Negative |
| 31 | 0.868 | Positive |
| 32 | 0.891 | Positive |
| 33 | 0.845 | Positive |
| 34 | 1.09 | Positive |
| 35 | 0.729 | Positive |
| 36 | 1.019 | Positive |
| 37 | 0.761 | Positive |
| 38 | 0.953 | Positive |
| 39 | 1.058 | Positive |
| 40 | 0.93 | Positive |
| 41 | 0.986 | Positive |
| 42 | 0.987 | Positive |
| 43 | 0.797 | Positive |
| 44 | 0.856 | Positive |
| 45 | 1.002 | Positive |
| 46 | 0.921 | Positive |
| 47 | 0.87 | Positive |
| 48 | 0.813 | Positive |
| 49 | 0.907 | Positive |
| 50 | 0.853 | Positive |
